# Racial and ethnic disparities in ED use among older adults with asthma and primary care nurse practitioner work environments

**DOI:** 10.21203/rs.3.rs-3972673/v1

**Published:** 2024-03-14

**Authors:** Lusine Poghosyan, Jianfang Liu, Eleanor Turi, Kathleen Flandrick, Marcia Robinson, Maureen George, Grant Martsolf, J. Margo Brooks Carthon, Monica O’Reilly-Jacob

**Affiliations:** Columbia University School of Nursing; Columbia University School of Nursing; Perelman School of Medicine, University of Pennsylvania; Columbia University School of Nursing; Columbia University School of Nursing; Columbia University; University of Pittsburgh; University of Pennsylvania; Columbia University School of Nursing

**Keywords:** nurse practitioner, work environment, older adults, emergency department, primary care

## Abstract

**Background:**

Nurse practitioners (NPs) increasingly deliver primary care in the United States. Yet, poor working conditions strain NP care. We examined whether racial/ethnic health disparities in ED visits among older adults with asthma are moderated by primary care NP work environments.

**Methods:**

Survey data on NP work environments in six states were collected from 1,244 NPs in 2018–2019. 2018 Medicare claims data from 46,658 patients with asthma was merged with survey data to assess the associations of all-cause and ambulatory care sensitive conditions (ACSC) ED visits with NP work environment and race/ethnicity using logistic regression.

**Results:**

NP work environment moderated the association of race (Black patients versus White patients) with all-cause (odds ratio [OR]: 0.91; p-value = 0.045) and ACSC (OR: 0.90; p-value = 0.033) ED visits.

**Conclusions:**

Disparities in ED visits between Black and White patients with asthma decrease when these patients receive care in care clinics with favorable NP work environments.

## Introduction

Over 4 million adults age 65 and older in the United States (U.S.) have an asthma diagnosis.^1^ Asthma is often undertreated and underdiagnosed in older adults, partly because its symptoms can be confused with other chronic conditions common in older adults, such as chronic obstructive pulmonary disease, interstitial lung disease, eosinophilic bronchitis, heart failure, bronchiectasis, and sarcoidosis.^2,3^ In addition, the physiological and psychological effects of aging can complicate the diagnosis, treatment, and management of asthma among older adults.^4,5^ Asthma is often successfully managed by primary care clinicians^6^; however, when patients lack access to available or high-quality primary care, they may experience asthma exacerbations and visit the emergency department (ED) for care.^7^ Older adults with asthma frequently visit the ED, at a rate of 18.6 visits per 10,000 population.^8^ This outcome is costly for the patient and the healthcare system and could be avoided via high-quality care in primary care settings.^7,9^

Within the population of older adults with asthma in the U.S., individuals from certain racially and ethnically minoritized groups experience disproportionately higher asthma prevalence, morbidity, and mortality.^10^ For example, Black and Hispanic adults are twice as likely as White adults to report an asthma-related ED visit in the last 12 months.^11^ Social and structural determinants of health contribute to asthma disparities, including issues related to inequitable access and quality of health care for Black and Hispanic adults.^12–15^ Health care system interventions are needed to address these disparities.^15^

Access to high-quality primary care can help older adults from racially and ethnically diverse backgrounds improve asthma control and thus reduce asthma-related health disparities. An increasing proportion of primary care delivered in historically marginalized and primary care underserved communities is provided by nurse practitioners (NPs).^16,17^ Nurse Practitioners are advanced practice registered nurses with either a Master’s or Doctoral degree.^18^ As licensed, independent clinicians, NPs can provide a wide range of health care services, including the diagnosis and management of diseases and health promotion and education. The NP workforce is experiencing rapid growth, reaching 385,000 NPs in 2023, with a projected 45% growth between 2022–2032.^19,20^ Research shows that NPs provide safe, high-quality primary care, and the outcomes of their care are comparable to those of physicians.^17,21^ Yet NPs face many working challenges within their clinical settings, which limits their capacity to deliver care to their patients. The work environment of NPs, comprised of NP perceptions of working conditions in their organizations, plays a key role in NP delivery of care and outcomes.^22^ A favorable NP work environment consists of collegiality between NPs and physicians, clear communication between NPs and administrators, understanding of the NP capabilities within the practice, and support for the NP independent practice.^23^ In unfavorable work environments, NPs have limited administrative support, strained relations with staff, and inequitable access to clinic resources such as staff support.^24,25^ For example, staff support is often unavailable to NPs to perform patient care activities, such as in-office screening and diagnostic testing or lab draws^26^, activities that are essential to achieving and sustaining asthma control.

Unfavorable NP work environments are also associated with a lower quality of patient care, a lack of patient-centered care, and increased hospitalizations and ED utilization.^25,27^ The NP Health Disparities model, which shows the relationship between various community, policy, and organizational factors affecting NPs and health disparities, posits that an unfavorable work environment exacerbates health disparities by limiting NP’s ability to deliver high-quality care to patients from historically marginalized communities in the U.S..^28^ Yet, it is unknown how the NP work environment contributes to disparities among older adults with asthma. As the population of older adults in the U.S. is becoming more racially and ethnically diverse and the costly burden of chronic conditions increases,^29,30^ evidence on the impact of the NP work environment on reducing racial health disparities in unnecessary acute care utilization in historically marginalized communities is needed. The purpose of this study was to examine whether racial and ethnic health disparities in ED visits among older adults with asthma are moderated by the NP work environment in primary care practices.

## Methods

### Design

We used a cross-sectional design to collect survey data on work environments from NPs in primary care practices in six U.S. states in 2018–2019. The survey data was merged with 2018 Medicare claims data for patients from the same practices. This study received approval from the Institutional Review Board of [removed for review] Medical Center. Below, we provide an overview of the methods presented in detail and published elsewhere.^25,31^

### Study Setting

We conducted the study in six geographically diverse states across the US: Arizona (AZ), California (CA), New Jersey (NJ), Pennsylvania (PA), Texas (TX), and Washington (WA). Each state in the U.S. has policies determining the care and services NPs can provide to their patients.^32^ These state-level scope of practice regulations determining NPs’ autonomy level in delivering patient care vary from state to state.^32^ NPs practicing in AZ and WA had a full scope of practice, they could treat patients and prescribe medications independently; in NJ and PA, they had a reduced scope of practice, NPs needed to collaborate with physicians to deliver care; and in CA and TX they had a restricted scope of practice, NPs required physician supervision to practice.

### Study Sample

#### Primary Care Practices

We defined primary care practices as those in which 50% or more of the physicians had family practice, general practice, geriatrics, internal medicine, preventative medicine, or pediatrics specialties. We then selected primary care practices that employed at least one NP for selection in our study.

#### Nurse Practitioner Sample

To identify NPs in these primary care practices, we used IQVIA’s OneKey database, which contains information on ambulatory-based clinicians in the U.S., including clinician and practice names, practice locations, contact information, and National Provider Identification (NPI) numbers.^33^ We requested the full sample of primary care NPs in AZ, NJ, and WA, a 75% random sample of NPs in PA, and a 50% random sample in CA and TX, given the difference in the NP workforce size across states, with CA and TX having a large number of NPs. Using a modified Dillman process^34^, we sent three separate survey mailings and two postcard reminders to collect data. Each mailing included an online link to the survey, giving NPs a choice to complete the survey online or on paper. In total, 5,689 NPs met the sampling criteria, and 1,244 NPs returned completed surveys (a response rate of 21.9%). More information on the survey, response rate, and nonresponse bias is reported elsewhere.^31^

#### Patient Sample

We first obtained Medicare claims for all patients (n = 1,123,861) who received care in study practices in 2018. Medicare is a federal insurance plan that offers insurance coverage to all adults 65 years and older. Patients were attributed to the practices where they received care using a common approach^35^ in which they were first attributed to clinicians (NPs and physicians) by NPI and then to a specific practice using the practice identifier from the OneKey database. Patients without a dominant clinician were excluded from the analysis (n = 642). We then selected only patients ages ≥ 65 who had been diagnosed with asthma based on the Centers for Medicare & Medicaid Services Chronic Condition Warehouse algorithm.^36^ Patients younger than 65 may also receive Medicare for certain qualifying reasons, including disability. Our final sample included 46,658 patients with asthma attributed to 917 primary care practices, for which we had both NP surveys and patient data.

### Variables

#### Key Explanatory Variables

We measure the explanatory variable, race and ethnicity, using the Research Triangle Institute (RTI) race and ethnicity coding algorithm, which included seven categories: African American or Black (Black), Asian or Pacific Islander (Asian), Hispanic or Latinx (Hispanic), non-Hispanic White (White), American Indian/Native American, Unknown, and Other. This algorithm improves the sensitivity of identifying patients from various racial and ethnic groups compared to the Medicare race variable alone.^37^

NPs completed the Nurse Practitioner-Primary Care Organizational Climate Questionnaire (NP-PCOCQ)—a reliable and valid measure of the NP work environment.^23^ The NP-PCOCQ contains 29 items asking NPs how much they agree that certain work conditions are present in their practices on a 4-point Likert-like scale from “strongly agree” to “strongly disagree”. The tool has four subscales measuring critical work environment domains: NP-Administration Relations (NP-AR), NP-Physician Relations (NP-PR), Independent Practice and Support (IPS), and Professional Visibility (PV).^23^ For example, the NP-AR subscale measures if practice managers take NP concerns seriously or make efforts to improve their working conditions, and the IPS subscale measures NPs’ ability to practice independently and have support for their practice. The NP-PCOCQ and its subscales have strong psychometric properties with acceptable internal consistency reliability and construct, discriminant, and predictive validity.^23,38^

We first computed individual-level mean scores on each NP-PCOCQ subscale. Then, we calculated practice-level mean scores by aggregating the responses of all NPs within each practice since the work environment is a property of the organization, not the individual NP.^39^ A global measure of practice-level work environment was computed by averaging scores of the four NP-PCOCQ subscales; higher scores indicate a more favorable work environment. We created a standardized work environment score for the regression models with an average score of 0 and a standard deviation of 1.

#### Outcome Variables

We measured two outcomes: all-cause and Ambulatory Care Sensitive Conditions (ACSC) ED visits, coded categorically (zero events or 1 or more events). Using Medicare Part B claims data, we identified all-cause ED visits as any visit for Healthcare Common Procedure Coding System codes 99281, 99282, 99283, 99284, and 99285.^40^ ACSC ED visits were unique ED visits with evidence of being avoidable or primary care treatable according to the “New York University ED Algorithm”.^41^ For each ED visit, the algorithm assigns a probability, based on the primary International Classification of Diseases (ICD) 10-CM diagnosis, that the visit is in one of five categories: 1- Non-Emergent; 2- Emergent, Primary Care Treatable; 3- Emergent, ED Care Needed, Preventable/Avoidable; 4- Emergent, ED Care Needed, Not Preventable/Avoidable; 5- All other. ED visits were counted as ACSC if the probability of belonging in the first three categories was greater than 0, based on the principal ICD-10-CM diagnosis from the Medicare Part B claims data.

#### Covariates

We included patient demographic information (i.e., age, sex) and the type of patients’ dominant primary care clinician (i.e., physician, NP) as patient-level covariates. In addition, because asthma and chronic obstructive pulmonary disease (COPD) are heterogeneous conditions that often overlap in older adults,^42^ we included having COPD separately, as well as the number of 13 chronic conditions from the U.S. Department of Health and Human Services list of standard chronic conditions (i.e., hypertension, congestive heart failure, coronary artery disease, cardiac arrhythmias, hyperlipidemia, stroke, arthritis, cancer, chronic kidney disease, dementia, depression, diabetes, osteoporosis).^43^ To fit the most parsimonious model, we counted chronic conditions. Additionally, the count of conditions is a better predictor of total Medicare expenditures than the cumulative duration of chronic conditions.^44^

At the organizational level, we controlled for the number of NPs and physicians in practice, practice type (i.e., physician offices, hospital-based clinics, community health centers, other), location (rural vs. urban), and practices’ structural capability index measuring the structural attributes (i.e., availability of the electronic health record, disease registries, weekend hours, performance feedback to clinicians, disease registries and reminder systems, community referrals, shared communication with patients) associated with the delivery of high-quality care.^45^ Fixed-effects for each state were included to account for differences at the state level, including scope of practice variation. Though missing data was < 5% of the survey data, we assessed patterns of missingness and found the missingness to be independent of NPs’ demographic attributes. Thus, case-wise deletion was used. There was no missing data in the Master Beneficiary Summary File, from which patients’ demographic information was extracted.

### Data Analysis

We computed descriptive statistics for all patient-level and organizational-level characteristics. Bivariate associations between patient-level characteristics (i.e., age, dominant primary care provider type, having COPD, number of other chronic conditions, and sex) and race/ethnicity (i.e., White, Hispanic, Black, Asian, and all other) were calculated using analysis of variance or chi-square tests. Frequencies of outcomes (i.e., all-cause and ACSC ED visits) by race and ethnicity group were also calculated. Finally, we used mixed-effect logistic regression models to assess the associations of outcomes with NP work environment and race and ethnicity, controlling for patient- and organizational-level factors as described above. An interaction term between race and ethnicity and work environment score was created to assess whether the work environment moderated racial and ethnic disparities. Next, we estimated the odds ratio of each outcome at different levels of NP work environment score (from − 2 to a maximum score of 1.60) from the final model, including the significant interaction effect to further examine how work environment impacted racial and ethnic disparities in ED and ASCS ED visits. To account for the clustering effect of 46,658 patients nested in 917 practices, we used random effect models and adopted a two-sided α level of .05. Our organizational-level sample size of 917 practices is greater than the recommended sample size of 50 at the second level to run random effect models.^46^ Our sample size allows us to detect a small effect size with 80% power. All analyses were conducted in SAS, version 9.4 (SAS Institute, Inc., Cary, NC).

## Results

### Characteristics of Patients

Overall, we included 46,658 patients age 65 and older who were diagnosed with asthma and had received care at one of the primary care practices in our study (Table 1). Patients had a mean age of 74.8 years (SD = 7.2), and 70.1% were women. Black patients had the highest average number of chronic conditions of all racial and ethnic groups, 3.7 conditions (SD = 2.2), and the highest proportion of COPD (39.3%). Asian patients were less likely to have an NP as their primary care clinician (10.4%) than all other race and ethnicity groups.

### Characteristics of Primary Care Practices

We included 917 primary care practices employing 1,244 NPs who participated in the survey (Table 2). On average, each practice served 667 patients with a diagnosis of asthma (SD = 861.8) and employed 2.8 NPs (SD = 3.8) and 6.2 physicians (SD = 15.2). Most practices were owned by physicians (54.4%) and located in an urban area (87%).

### All-Cause and ACSC ED Visits

In total, 16,478 patients with asthma (35.3%) visited the ED, and 11,644 patients with asthma (25%) had an ACSC ED visit (Table 1). Black and Hispanic patients were more likely than White patients to both have an all-cause (Black 48.1%; Hispanic 44%; White 33.9%) and ACSC ED visits (Black 38.3%; Hispanic 33.6%; White 23.4%).

#### Moderation Effects of NP Work Environment on Racial and Ethnic Disparities in ED Visits among Patients with Asthma

**Figure 1:**
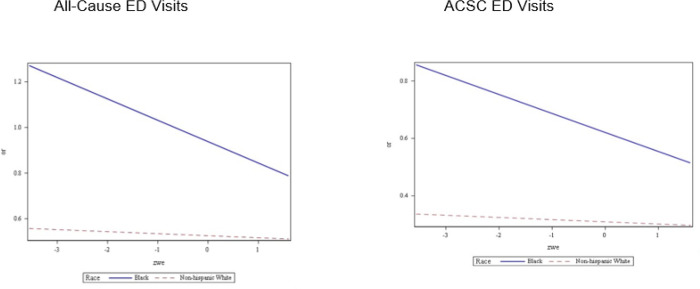
Odds of All-Cause and ACSC ED Visits among Medicare Patients with Asthma by Race and Standardized NP Work Environment Score (n=46,658) Abbreviations: NP = nurse practitioner, ACSC = ambulatory care sensitive condition, ED = emergency department, OR = odds ratio

We found that NP work environment in primary care practices moderated the association of race (Black patients versus White patients) with all-cause (odds ratio [OR]: 0.91; p-value = 0.045) and ACSC (OR: 0.90; p-value = 0.033) ED visits among Medicare patients with asthma (Table 3). Greater standardized work environment scores were associated with lower odds of all-cause and ACSC ED visits between Black and White patients (Supplementary Table 1). When the standardized NP work environment measure reached its maximum score of 1.60—indicating the most favorable environment, the odds ratio for all-cause and ACSC ED visits for Black patients compared to White patients were the lowest (all-cause ED visits 1.42; 95% confidence interval [CI] = 1.18–1.70; p-value = 0.002 and ACSC ED visits 1.57; 95% CI 1.29–1.90; p-value < 0.001). Conversely, when the standardized work environment measure reached its lowest score of −2, the odds of all-cause and ACSC ED visits for Black patients compared to White patients were their highest (all-cause ED visits 1.97; 95% CI 1.64–2.36; p-value < 0.001 and ACSC ED visits 2.25; 95% CI 1.87–2.72; p-value < .001) (Supplementary Table 1, Fig. 1). As the work environment scores increased, both White and Black patients with asthma had lower odds of all-cause or ACSC ED visits (Fig. 1).

Compared to White patients with asthma, we found that the NP work environment did not moderate the associations between other race and ethnicity groups (i.e., Asian and Hispanic patients) and all-cause or ACSC ED visits. Overall, Asian patients were equally likely to have an all-cause or ACSC ED visit compared to White patients. Hispanic patients were more likely to experience both all-cause (OR 1.50; 95% CI 1.38–1.62; p-value < 0.001) and ACSC (OR 1.63; 95% CI 1.50–1.78; p-value < 0.001) ED visits compared to White patients.

## Discussion

In this study, we examined whether NP work environments in primary care practices affect racial and ethnic disparities in ED visits for older adults with asthma. Our findings show a significant disparity; one in four White patients with asthma had at least one all-cause ED visit, while almost half of the Black patients with asthma (48%) had an all-cause ED visit. Similarly, the rates were higher for Black and Hispanic patients for ACSC ED visits, deemed preventable with high-quality primary care. Our findings also show that disparities in ED visits between Black and White patients with asthma decrease when these patients receive care in primary care practices with favorable NP work environments.

Results are consistent with previous findings that, overall, better NP work environments improve quality outcomes, enhance patient-centeredness, and reduce hospitalizations and ED utilization.^25,27^ Our study findings, however, add that not all patient groups experience this benefit—and in fact, Black patients are disproportionately *more* likely to experience improved outcomes when the NP work environment is more favorable. Over the years, many interventions to reduce racial and ethnic disparities have been tested in the U.S.^47,48^, with an overall lack of robust effect. The results of this study suggest that improving the work environments of NPs in primary care may be an effective tool to address disparities in asthma outcomes.

Preventing unnecessary ED visits among older adults with asthma is a likely benefit of favorable NP work environments. NPs practicing in favorable work environments may be more likely to provide evidence-based care to their patients with asthma. In such environments, NPs also have adequate support and resources, favorable collegial relationships, and optimal use of their advanced skills.^25,27^ Thus, supporting favorable NP work environments may help patients receive high-quality care and prevent ED visits.

Also, asthma control can be improved when clinicians and patients engage in shared decision-making.^49^ This is important because other interventions to improve asthma control, such as coaching^50^, have not been effective. NPs may be particularly well-suited to be engaged in shared decision-making as their education typically includes training in therapeutic communication strategies and patient engagement. Also, NP practice is characterized as more holistic in its orientation to care, allowing NPs to develop trusting relationships with their patients as they design treatment plans to accommodate the care that patients want and value. Favorable work environments may promote NPs’ ability to engage in shared decision-making, translating it into better patient outcomes.

Despite the positive attributes associated with NP care, significant barriers to asthma control persist. Without access to high-quality care, community resources, and pharmacologic treatments, patients will likely continue to suffer from poor asthma control that places them at risk for reduced quality of life, future acute health care utilization, and even death. While increased healthcare coverage remains out of reach for many Americans, NPs can have an immediate effect by delivering high-quality, patient-centered care to the communities that need it most, given that their numbers are growing rapidly. Our findings present critically important policy and practice implications in reducing racial and ethnic healthcare disparities. As the NP workforce grows, they are vital to delivering care in communities underserved by primary care and helping narrow the health disparity gap. Yet, despite the importance of the NP work environment, many NPs report practicing in challenging environments that do not provide adequate time and resources for patient care. Investing in work environments to allow NPs to deliver high quality care is critically needed for the goal of eliminating racial and ethnic health care disparities in the U.S.

Our findings also may have international implications. The healthcare workforce globally has undergone major changes in recent decades triggered by a growing prevalence of chronic conditions, shortage of primary care providers, and advancement of nursing education.^51,52^ As a result, countries increasingly rely on nurses with advanced roles, such as NPs.^53^ In many countries, policies are being designed to shift tasks from physicians to advanced practice nurses as an effective strategy to increase primary care capacity, meet the demand for care, and achieve the goals of universal health coverage.^54^ Thus, creating favorable work environments for this growing workforce should be a priority to achieve these goals.

Our study has limitations. We collected data in six U.S. states with a variable NP scope of practice regulations, so the findings may not be generalizable nationwide. Future studies should be expanded nationally. Our findings may also not be generalizable internationally given the different demographic distribution and healthcare system characteristics in the U.S. Future international studies should be conducted, given the rapid growth of NPs internationally. Some patients with asthma in the study also likely had COPD. While we adjusted separately for COPD, it can be challenging to distinguish clinically between COPD and asthma in older adult populations, given that the two conditions are heterogeneous airway diseases that often overlap.^55^ We relied on self-reported data from NPs, which are subject to self-report bias. Our about 22% response rate is low yet comparable to other clinician survey responses.^56^ Therefore, non-response bias might be an issue, yet responders were not significantly different from non-responders, and the demographic characteristics of our sample were comparable to those in the national NP sample.^31^ Our study used a cross-sectional, observational design, and determining causation is not possible. While this study produces important insights on the potential impact of NP work environments on the outcomes of patients with asthma, more research is needed to understand better the aspects of the work environment that can enhance the quality of asthma care delivery. Furthermore, intervention studies should be designed to determine the impact of work environment on ED use among patients with asthma.

## Conclusion

This is the first study that investigated the association between NP work environment in primary care practices and racial and ethnic health disparities in ED use among older adults with asthma in the U.S. Merging unique survey and patient data, we found that the NP work environment moderates the relationship between race and ethnicity and ED use. When patients receive care in practices with poor NP work environments, Black older adults are even more likely to have higher rates of all-cause and ACSC ED visits compared to White patients. Our findings have key implications for practice, policy, and research innovations to enhance the NP work environment in primary care practices and achieve health equity.

## Figures and Tables

**Table 1:** Patient Characteristics by Race and Ethnicity Groups

Characteristic		Race and Ethnicity, (n, %)					
	Total (N = 46,658)	Asian (N = 1,471)	Black (N = 2,546)	Hispanic (N = 3,300)	Non-Hispanic White (N = 37,861)	Other^a^ (N = 1,480)	P-Value
Age, mean (SD)	74.8 (7.2)	76.2 (7.7)	73.6 (7.1)	74.5 (7.3)	74.93 (7.19)	72.09 (6.01)	< 0.001
No. of chronic conditions, mean (SD)	3.2 (2.1)	3.3 (2.1)	3.7 (2.2)	3.6 (2.2)	3.1 (2.1)	2.8 (2.0)	< 0.001
Sex							
Female	32,719 (70.1)	989 (67.2)	1,973 (77.5)	2,424 (73.4)	26,439 (69.8)	894 (60.4)	< 0.001
Male	13,939 (29.9)	482 (32.8)	573 (22.5)	876 (26.5)	11,422 (30.2)	586 (39.6)	
Comorbidity							
COPD	17,008 (36.4)	508 (34.5)	1,001 (39.3)	1,170 (35.4)	13,925 (36.8)	404 (27.3)	< 0.001
Type of PCP							
Physician	38,563 (82.6)	1,318 (89.6)	2,125 (83.5)	2,701 (81.8)	31,222 (82.5)	1,197 (80.9)	< 0.001
NP	8,095 (17.3)	153 (10.4)	421 (16.5)	599 (18.1)	6,639 (17.5)	283 (19.1)	
Patient outcomes							
ED visits	16,478 (35.3)	479 (32.6)	1,225 (48.1)	1,452 (44.0)	12,843 (33.9)	479 (32.4)	< 0.001
ACSC ED visits	11,644 (25.0)	370 (25.1)	975 (38.3)	1,109 (33.6)	8,846 (23.4)	344 (23.2)	< 0.001

COPD = chronic obstructive pulmonary disease; PCP = primary care provider; NP = nurse practitioner ED = emergency department; ACSC = ambulatory care sensitive condition

a Includes: American Indian/Alaskan Native, Unknown Race and Ethnicity, and Other Race and Ethnicity

**Table 2:** Organizational-level Characteristics of Primary Care Practices Employing NPs

Characteristics	Value (N = 917)
Practice size (mean/SD)	
Number of patients	667.0 (861.8)
Number of NPs	2.8 (3.8)
Number of physicians	6.2 (15.2)
NP Work Environment score (mean/SD)	3.2 (0.5)
Structural capability index score (mean/SD)	0.6 (0.2)
Location, n (%)	
Rural	119 (13.0)
Urban	798 (87.0)
Practice type, n (%)	
Community health center	125 (13.8)
Hospital-based clinic	90 (9.9)
Physician practice	493 (54.4)
Other/Unsure	199 (21.9)
States, n (%)	
Arizona	99 (10.8)
California	197 (21.5)
New Jersey	97 (10.6)
Pennsylvania	223 (24.3)
Texas	189 (20.6)
Washington	112 (12.2)

Percentages may not total 100 because of rounding.

NP = nurse practitioner

**Table 3:** Multi-level Regression Models Assessing the Moderation Effect of NP Work Environment on Associations of Race and Ethnicity with All-Cause and ACSC ED Visits among Medicare Patients with Asthma (n = 46,658)

	All-Cause ED Visits^c^		ACSC ED Visits^c^	
Race and Ethnicity	OR (95% CI)	P-value	OR (95% CI)	P-value
Asian	0.90 (0.79–1.02)	0.097	1.09 (0.95–1.24)	0.226
Black	1.64 (1.50–1.79)	< .001***	1.841 (1.68–2.02)	< .001***
Hispanic	1.49 (1.38–1.62)	< .001***	1.634 (1.50–1.78)	< .001***
Non-Hispanic White	Ref	Ref	Ref	Ref
Other^a^	1.06 (0.94–1.20)	0.304	1.108 (0.97–1.26)	0.124
Work Environment^b^	0.98 (0.95–1.02)	0.324	0.975 (0.94–1.01)	0.179
Asian Race x Work Environment	0.96 (0.85–1.08)	0.484	1.020 (0.90–1.16)	0.755
Black Race x Work Environment	0.91 (0.83–0.99)	0.045*	0.904 (0.82–0.99)	0.033*
Hispanic Race x Work Environment	0.97 (0.89–1.05)	0.434	0.991 (0.91–1.08)	0.836
Non-Hispanic White Racewx Work Environment	Ref	Ref	Ref	Ref
Other Race x Work Environment	1.03 (0.91–1.16)	0.628	1.089 (0.95–1.24)	0.202

* P value less than .05;

** P value less than .01;

*** P value less than .001

## Data Availability

The data supporting this study’s findings are available from the corresponding author upon request. All data provided will be anonymized, and terms for usage will be agreed in advance.
